# Extra-anatomical Bypass in a Case of Recoarctation and Previous Cardiac Surgery

**DOI:** 10.7759/cureus.69444

**Published:** 2024-09-15

**Authors:** Assen Keltchev, Kristiyanna Mavrodieva, Andrey Neutov, Evgeni V Mekov, Georgi Yankov

**Affiliations:** 1 Department of Cardiosurgery, Acibadem City Clinic, University of Sofia, Sofia, BGR; 2 Department of Pulmonary Diseases, Medical University - Sofia, Sofia, BGR

**Keywords:** aortic recoarctation, aortic stenosis, cardiac surgery, extra-anatomic bypass, resternotomy

## Abstract

A complex clinical case of aortic recoarctation is presented. The case is a 61-year-old comorbid patient with two previous aortic and cardiac operations. At the age of 10, the patient underwent surgery for post-ductal coarctation of the aorta (adult type) at the typical site, where the stenotic area was completely resected, and an end-to-end anastomosis was performed through a left-sided thoracotomy. Ten years ago, the patient also had mitral valve replacement with a metallic prosthesis due to severe mitral insufficiency, performed via median sternotomy. The patient was admitted to the cardiac surgery unit due to symptoms suggestive of aortic re-stenosis due to status post repair of coarctation of the aorta (resection with end-to-end anastomosis). An extra-anatomic bypass was performed between the ascending and abdominal aorta, with the graft passing through a new diaphragm opening in front of the hilus of the right lung.

## Introduction

Coarctation of the aorta (CoA) is a congenital narrowing of the aortic lumen, typically occurring distal to the origin of the left subclavian artery. It represents 5% to 7% of all congenital heart defects and occurs in approximately one in 2500 live births [1,2]. This stenosis can lead to a significant pressure gradient between the upper and lower body, causing hypertension in the upper extremities and diminished perfusion in the lower extremities. If left untreated, CoA can result in long-term complications, including left ventricular hypertrophy, heart failure, aortic rupture, stroke, and other cardiovascular diseases.

Surgical repair is considered the primary method of treatment, particularly in infants and children, to prevent complications associated with prolonged hypertension. Techniques include resection of the stenotic segment with end-to-end anastomosis or patch aortoplasty. In older children and adults, stenting has become a less invasive option, offering effective outcomes with reduced recovery time [3].

This case report presents a detailed clinical case of aortic recoarctation following initial surgical repair.

## Case presentation

A 61-year-old man was hospitalized in a heart surgery clinic with complaints for six months of fatigue and shortness of breath on minimal exertion and weak intermittent pain in the lower extremities after exertion. He reported a medical history of arterial hypertension, cerebrovascular incident (a previous ischemic stroke in the precerebral arteries), dyslipidemia, hyperuricemia, and hypothyroidism. At the age of 10 years, the patient was operated on for post-ductal coarctation of the aorta, adult type at a typical site to complete obturation, the stenotic area was resected, and an end-to-end anastomosis was performed through a left-sided thoracotomy. Due to severe mitral insufficiency, mitral valve replacement with metallic prosthesis Sorin Carbomedics 29 (Milan, Italy) was performed via median sternotomy 10 years ago, with a cardiopulmonary bypass (CPB) time of 78 minutes and an aortic cross-clamp time of 42 minutes. On physical examination, the blood pressure was measured as 150/70 mmHg on the right radial artery and left radial artery, and 94/38 mmHg on the right femoral artery. All other parameters and lab tests were normal.

**Figure 1:**
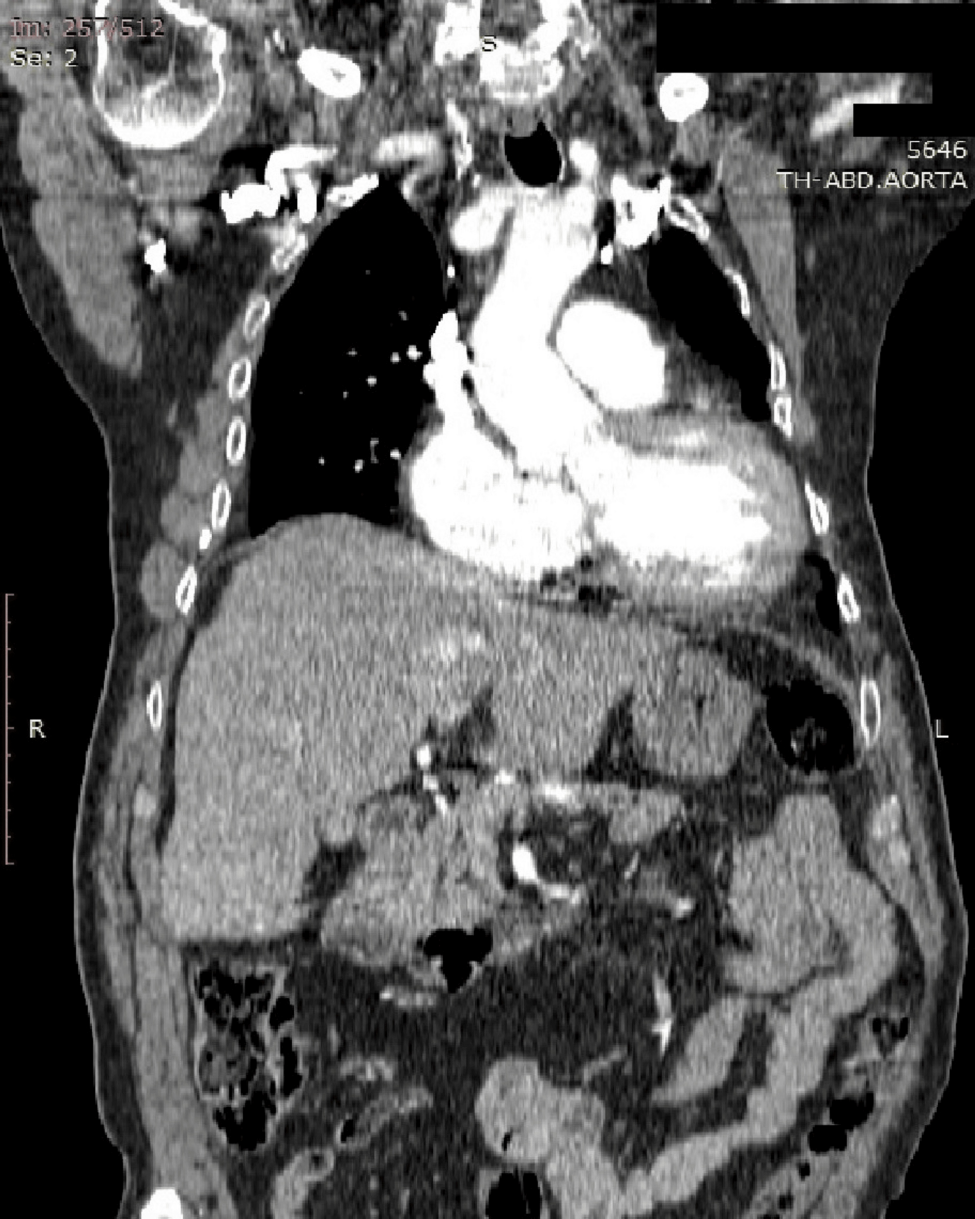
Preoperative coronal computed tomography view of the patient.

A computed tomography scan of the thoracic and abdominal aorta revealed aortic stenosis at the level of the aortic arch, with a narrowing to 0.76 cm after the left subclavian artery (Figure 1). Echocardiography revealed a descending aorta with a preoperative abnormal peak/mean gradient of 56/32 mmHg after the left subclavian artery, which was reduced to no detectable gradient after the surgery.

**Figure 2:**
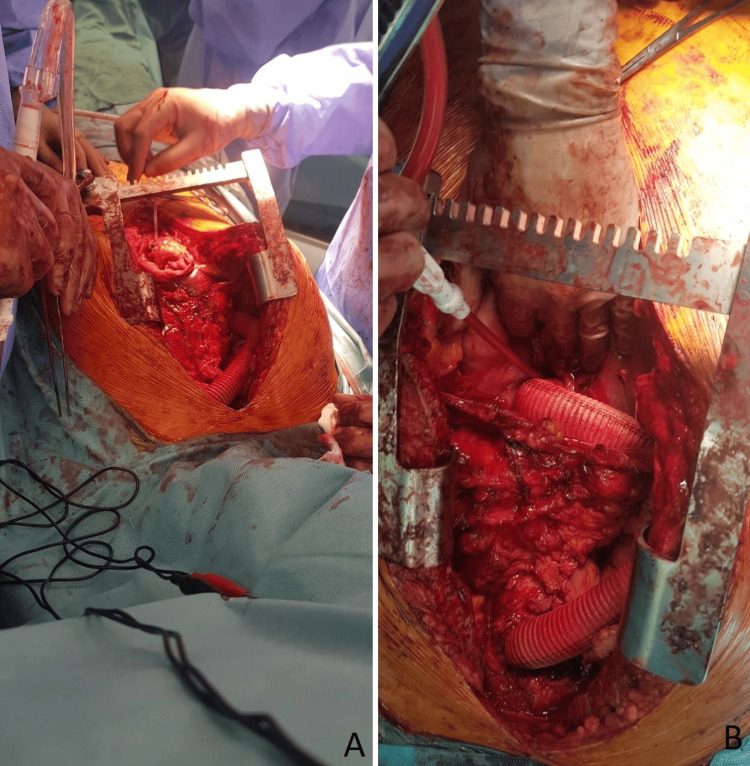
Intraoperative view of the placed vascular prosthesis for the implementation of the extra-anatomical bypass between the ascending aorta and the abdominal aorta. (A) View of the distal anastomosis from the laparotomy. (B) View of the proximal anastomosis from the sternotomy (in both images, left is patient's left, down is cephalic).

Vitamin K antagonist (3 mg daily, with the target international normalized ratio maintained between 2.5 and 3.5) was stopped five days before surgery and nadroparin 2 x 0.6 ml was started. Standard longitudinal resternotomy was performed. Severe pericardial adhesions were found, which were released in a predominantly acute fashion. In case of injury during the resternotomy, the backup plan for cannulation included the right axillary artery and the right femoral artery as alternative sites for perfusion. The incision was extended distally to an upper median laparotomy. The left lobe of the liver was mobilized. The right dome of the diaphragm was transected with a vertical incision and the beginning of the abdominal aorta was reached. No switch to CPB, cardiac arrest, or circulatory hypothermic arrest was required. The prerenal abdominal aorta was clamped inferiorly just above the celiac trunk and distal anastomosis was performed with the 22 mm vascular prosthesis and the latter using a wrapped 4/0 prolene suture of the end-to-side type. During the intervention, the circulation of the lower limbs was not compromised, and the arterial pressure monitored with an arterial cannula in the left radial and right femoral arteries remained stable and within normal limits. The adnexal flap was removed and the prosthesis was clamped. A vertical elliptical diaphragmotomy was performed, and the prosthesis was passed into the right pleural cavity, anterior to the hilus of the right lung. After the placement of a wall flap over the ascending aorta, proximal anastomosis was performed between the vascular prosthesis and the ascending aorta using a wrapped 4/0 prolene suture in an end-to-side fashion (Figure 2). Drains were placed retrosternally in the left pleural cavity and intra-abdominally.

**Figure 3:**
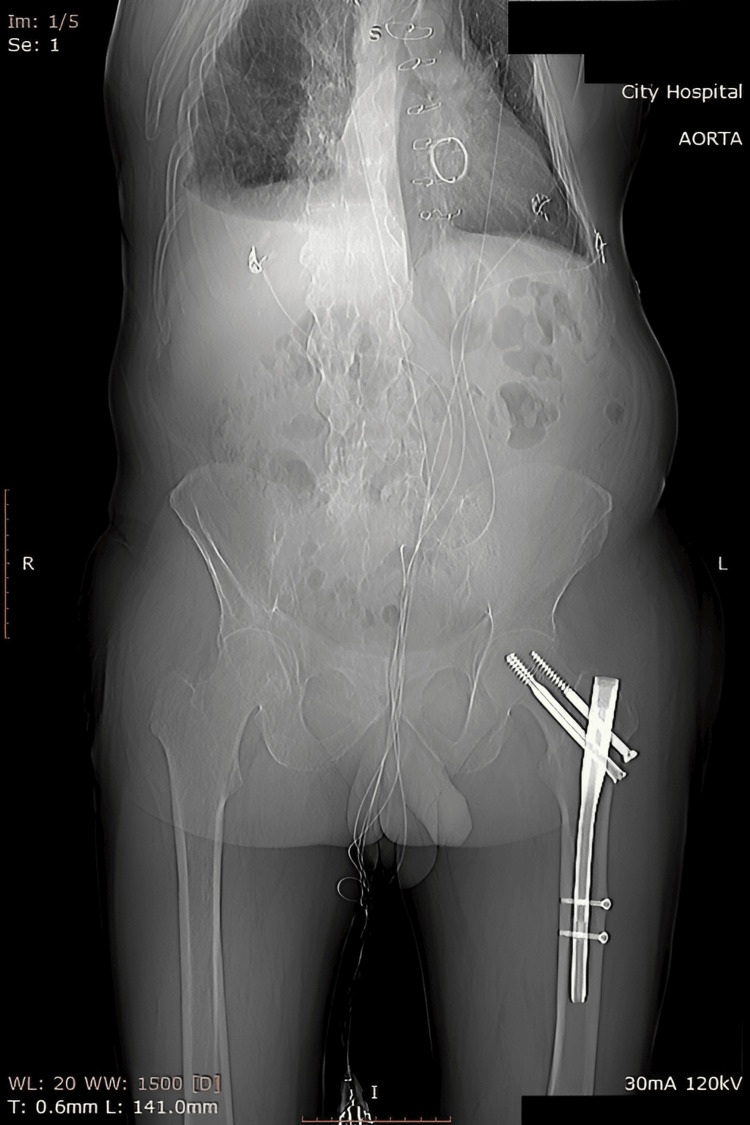
Postoperative X-ray of the patient.

**Figure 4:**
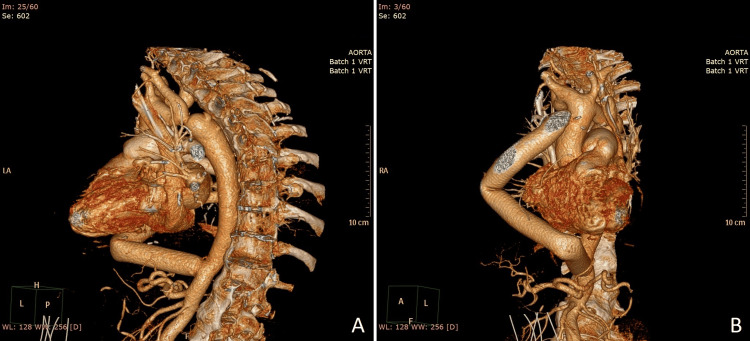
Postoperative CT angiography demonstrating the patency of the extra-anatomic bypass. (A) View of the distal anastomosis. (B) View of the proximal anastomosis.

The postoperative period was uneventful and without complications. Drains were removed on the second postoperative day and early rehabilitation was started. A postoperative X-ray of the patient (Figure 3) at discharge showed a hemodynamically insignificant pleural effusion, estimated at approximately 300 ml on ultrasound. The patient's postoperative CT scan (Figure 4) showed an extra-anatomic bypass between the ascending and abdominal aorta, with the filling of the graft with contrast material, which was patent and with angulations present. The supraaortal and visceral arteries were contrasted without evidence of stenoses. A variety of two left renal arteries and a double superior vena cava were also noted.

The patient was discharged on the eighth postoperative day without complications. After a one-year follow-up, no pathological gradient was observed.

## Discussion

This case report describes extra-anatomic bypass in recoarctation and previous cardiac surgery. Extra-anatomic bypass is a relevant option in cases with aortic recoarctation [4]. We also considered this technique as the patient had undergone two interventions, one for aortic coarctation by left thoracotomy, and the second on the heart by median sternotomy. Translating the prosthesis and anastomosis into the left pleural cavity with the descending aorta would have been significantly more difficult. The presence of adhesions, combined with the percutaneous thoracotomy, would have also posed an unreasonably higher risk of complications. For this reason, a resternotomy with an upper median laparotomy was performed, a new opening of the diaphragm was created to bring the graft in front of the hilus of the right lung, and a bypass was performed between the ascending and abdominal aorta. An adequate prosthesis bed was also created in front of the right hilus to prevent compression from surrounding structures. In such cases, massive adhesions are expected to be found and the perioperative risk of complications such as involvement of the ascending aorta, right atrium, right ventricle, and hemorrhage is increased [5].

Two randomized trials have shown the advantages of surgical technique over balloon dilatation in the primary treatment of the disease [6,7]. Both aortic restenosis and aortic aneurysm were observed as late complications. Aortic restenosis is relatively common, occurring in about 10% to 41% of cases, depending on the technique initially used in the previous coarctation repair and on patient characteristics: age, diameter of the segment with coarctation, and hypoplasia of the isthmus [8,9]. The need for reintervention due to the occurrence of late complications is seen in 5% to 50% of cases. The indications for correction in restenosis are similar to those in native coarctation: persistent arterial hypertension, peak gradient greater than 20 mmHg, presence of left ventricular hypertrophy, and presence of aortic aneurysm or collateral circulation on imaging [10].

## Conclusions

Surgical treatment remains the primary option for aortic coarctation, especially in childhood, with stenting serving as a viable alternative in selected cases. Reoperative surgical intervention for aortic recoarctation, however, carries a higher risk of complications. Extra-anatomic aortic bypass has proven to be an effective and reliable option in carefully selected patients with complex forms of primary or recurrent aortic narrowing.
